# The Pretreatment Glucose-to-Lymphocyte Ratio as an Independent Prognostic Biomarker in Ovarian Cancer

**DOI:** 10.3390/jcm15051999

**Published:** 2026-03-05

**Authors:** Ece Baydar, Yasemin Bakkal Temi, İlkay Çıtakkul, Devrim Çabuk, Umut Kefeli, Kazım Uygun

**Affiliations:** Department of Internal Medicine and Medical Oncology, Kocaeli University, 41380 Kocaeli, Turkey; yasemin.temi@kocaeli.edu.tr (Y.B.T.); ilkay.citakkul@kocaeli.edu.tr (İ.Ç.); devrimcabuk@yahoo.com (D.Ç.); ukefeli@yahoo.com (U.K.); kzuygun@hotmail.com (K.U.)

**Keywords:** glucose-to-lymphocyte ratio, epithelial ovarian carcinoma, prognosis, survival, biomarker

## Abstract

**Background/Objectives**: This study aimed to assess the prognostic significance of the glucose-lymphocyte ratio (GLR) prior to therapy in individuals with epithelial ovarian cancer. **Methods**: This retrospective cohort study included 326 patients with epithelial ovarian cancer who were treated from 2011 to 2025. The GLR was computed utilizing pre-treatment fasting blood glucose levels and absolute lymphocyte numbers. The optimal GLR cutoff value was established by receiver operating characteristic (ROC) analysis. Overall survival (OS) and disease-free survival (DFS) were assessed utilizing Kaplan–Meier analysis and Cox regression models. Additional sensitivity analyses were performed excluding patients with diabetes mellitus and by testing the interaction between GLR and neoadjuvant chemotherapy. **Results**: The optimal GLR cutoff value was 3.42. Patients were classified into low-GLR (≤3.42; *n* = 190) and high-GLR (>3.42; *n* = 136) groups. Patients with high GLR levels (>3.42) had a median OS of 58 months, which was significantly shorter than the 151 months for patients with low GLR levels (≤3.42) (*p* < 0.001). They also had a median DFS of 17 months, which was significantly shorter than the 49 months for patients with low GLR levels (*p* < 0.001). Multivariable Cox regression analysis showed that a higher GLR is an independent prognostic factor related to shorter overall survival (HR: 1.561; 95% CI: 1.078–2.261; *p* = 0.018). Findings remained consistent after excluding patients with diabetes mellitus. The group with a high GLR had a greater rate of disease progression (55.1% vs. 29.5%, *p* < 0.001). **Conclusions**: The pre-treatment GLR may serve as a simple and readily available prognostic biomarker in epithelial ovarian cancer, potentially supporting basic risk stratification; however, external validation is required.

## 1. Introduction

Ovarian cancer constitutes a major global health issue and continues to be a primary cause of death among gynecological cancers globally. GLOBOCAN 2022 estimates indicate roughly 324,000 new cases and over 206,000 deaths from ovarian cancer worldwide, highlighting its significant impact among cancers affecting women [1]. Epithelial ovarian cancer is the primary histological subtype, representing about 90% of all ovarian cancer cases, and is distinguished by significant biological and clinical heterogeneity, which complicates prognostic evaluation and treatment decision-making [2]. Despite advances in surgical techniques and systemic therapies, population-based statistics indicate that survival rates remain suboptimal, with an approximate 5-year relative survival rate of 50%, even in advanced healthcare systems [3]

Clinical and translational research indicates that the development and prognosis of ovarian cancer are intricately associated with systemic inflammatory responses that are evaluated using hematological indicators derived from standard blood tests [4]. Increased inflammation-based markers are associated with advanced disease stages and worse survival in ovarian cancer patients, with meta-analyses indicating that inflammatory ratios are elevated in malignant circumstances and correspond with disease progression [5,6]. Ovarian cancer demonstrates considerable metabolic reprogramming, characterized by heightened glucose use that interacts with inflammatory pathways to facilitate tumor growth and treatment resistance [7]. Inflammation is a crucial element of ovarian cancer biology, facilitating the advancement of inflammation-derived prognostic biomarkers [8]. Consistent data demonstrates that inflammation- and nutrition-related biomarkers obtained from standard laboratory parameters, such as the systemic immune–inflammation index (SII), the platelet-to-lymphocyte ratio (PLR), the prognostic nutritional index (PNI), and the modified Glasgow prognostic score (mGPS), have shown prognostic value in ovarian cancer populations [9,10,11].

The glucose-to-lymphocyte ratio (GLR) has emerged as a new biomarker that simultaneously indicates tumor-related glucose metabolism and systemic immunological function by combining two essential biological processes involved in cancer progression [12]. Hyperglycemia has been shown to facilitate tumor proliferation, treatment resistance, and diminished survival across multiple malignancies, while lymphopenia signifies compromised antitumor immune surveillance, thereby providing a compelling biological justification for integrating these parameters into a singular index. Despite the growing evidence, the prognostic significance of GLR in ovarian cancer remains largely unexplored. Most current research in this domain has concentrated on inflammation-based or nutrition-related indicators, neglecting the inclusion of metabolic factors in their analysis. This indicates a gap in the literature.

This study seeks to evaluate the prognostic value of the pretreatment GLR in ovarian cancer patients, considering the intricate relationship between glucose metabolism, systemic inflammation, and tumor progression, as well as the limited evidence regarding the GLR in this context. We sought to examine the correlation between the GLR and survival outcomes and to ascertain if the GLR could function as a straightforward, accessible, and clinically pertinent prognostic biomarker in this patient cohort [7,13].

## 2. Materials and Methods

This retrospective, single-center cohort research consecutively enrolled patients with histologically confirmed epithelial ovarian cancer treated at Kocaeli University Hospital from December 2011 to April 2025. This study complied with the principles of the Declaration of Helsinki and received clearance from the Kocaeli University Institutional Ethics Committee (approval number: GOKAKEK-2024/14/20, Project Identifier: 2025/337, approval date: 18 June 2025). Due to the study’s retrospective design, the ethics committee exempted the necessity for written informed consent.

### 2.1. Criteria for Inclusion and Exclusion

Eligible participants consisted of women aged 18 years or older with histologically confirmed epithelial ovarian cancer. The inclusion criteria included individuals who had received either primary cytoreductive surgery or interval debulking surgery after standard neoadjuvant chemotherapy. Pre-treatment serum glucose levels and absolute lymphocyte counts were mandated to be accessible, collected at the time of diagnosis, before the commencement of neoadjuvant chemotherapy or primary surgery. The exclusion criteria included a history of concomitant malignancies, inadequate clinical or laboratory data, and administration of non-standard or experimental neoadjuvant therapy regimens. The standard treatment was characterized as a chemotherapy regimen based on paclitaxel and carboplatin.

### 2.2. Data Collection

Data regarding age and the existence of diabetes mellitus, characterized by recorded medical history or current antidiabetic therapy, were gathered. Furthermore, clinical parameters including Federation of Gynecology and Obstetrics (FIGO) stage, histological subtype, tumor grade, date of diagnosis, date of surgery, progression or recurrence status, and date of the last visit or death were gathered. Tumor stage was recorded and re-coded according to the FIGO 2014 staging system (effective 1 January 2014), based on surgical and pathological reports. Given the long study period, temporal changes in supportive care and practice patterns may have occurred; however, all patients were managed according to contemporary standard-of-care protocols at our institution. Laboratory findings, including glucose levels, lymphocyte count, lactate dehydrogenase (LDH), and albumin levels, together with treatment-related data, were extracted from the institution’s electronic medical record system for each patient.

### 2.3. Definitions of Outcomes

Overall survival (OS) was the period between diagnosis and death from any cause or the date of the last follow-up. Disease-free survival (DFS) was defined as the time from the date of cytoreductive surgery (either primary debulking surgery or interval debulking surgery) to the first documented recurrence or progression, or the date of last follow-up. For patients treated with neoadjuvant chemotherapy, DFS was calculated starting from the date of interval debulking surgery, consistent with the definition applied to patients undergoing primary debulking surgery.

The calculation of GLR, the primary prognostic variable of interest in this study, was performed using the formula GLR = fasting blood glucose (mmol/L)/absolute lymphocyte count (×10^9^/L), alongside survival outcomes. Using a standard receiver operating characteristic (ROC) curve analysis for overall survival, the optimal cut-off value for GLR was determined using the Youden index (maximizing [sensitivity + specificity − 1]). Then, patients were split into high- and low-GLR groups.

### 2.4. Statistical Analysis

Statistical analyses were performed using IBM SPSS Statistics 29.0 (IBM Corp., Armonk, NY, USA). The distribution of the data was evaluated through the Kolmogorov–Smirnov and Shapiro–Wilk tests. Continuous variables are presented as the mean ± standard deviation or the median with the interquartile range, and categorical variables are expressed as frequencies and percentages. Comparisons between groups were performed using *t*-tests for normally distributed data and the Mann–Whitney U test for non-normally distributed data. Associations between categorical variables were assessed using the chi-square or Fisher’s exact test. ROC curve analysis was used to determine the optimal GLR cut-off for predicting OS. The cut-off was determined by the Youden index. Survival outcomes were examined using the Kaplan–Meier method, with comparisons made using the log-rank test. Independent prognostic factors were identified using univariate and multivariate Cox regression analyses. Variables deemed clinically relevant or statistically significant in the univariate analyses were incorporated into multivariate models. Hazard ratios (HRs) are presented with 95% confidence intervals (CIs), and a *p*-value of less than 0.05 was considered statistically significant. Internal validation of the GLR cut-off was performed using a 70%/30% training–validation split-sample approach. A sensitivity analysis was performed by excluding patients with diabetes mellitus. An interaction term between GLR and neoadjuvant chemotherapy status was included in the multivariate Cox regression model to assess potential effect modification.

## 3. Results

In a retrospective analysis of 326 ovarian cancer patients, the median age was 55 (IQR: 49–64). The median follow-up duration was 43 months (IQR: 24–72). 28% of the study population consisted of patients with stage I–II disease, while 71% consisted of patients with stage III–IV disease. A total of 288 patients (88%) underwent primary cytoreductive surgery, while 38 patients (12%) underwent interval debulking surgery after neoadjuvant therapy. The baseline characteristics of the patients are presented in Table 1.

**Figure 1:**
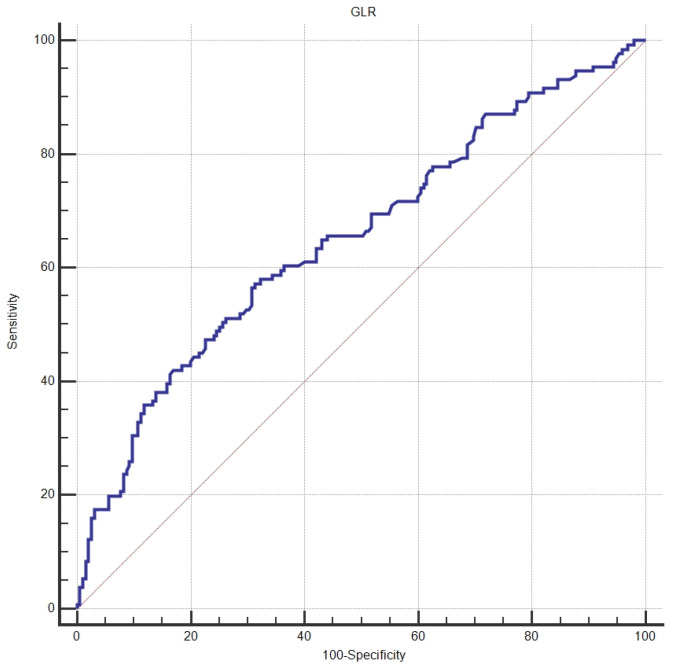
ROC curve demonstrating the discriminative performance of the GLR for survival status (AUC = 0.652; 95% CI, 0.597–0.703).

ROC analysis determined the optimal cutoff value for the glucose-to-lymphocyte ratio (GLR) to be 3.42 (Figure 1). Consequently, patients were categorized into low-GLR (≤3.42; *n* = 190, 58.3%) and high-GLR (>3.42; *n* = 136, 41.7%) groups.

**Figure 2:**
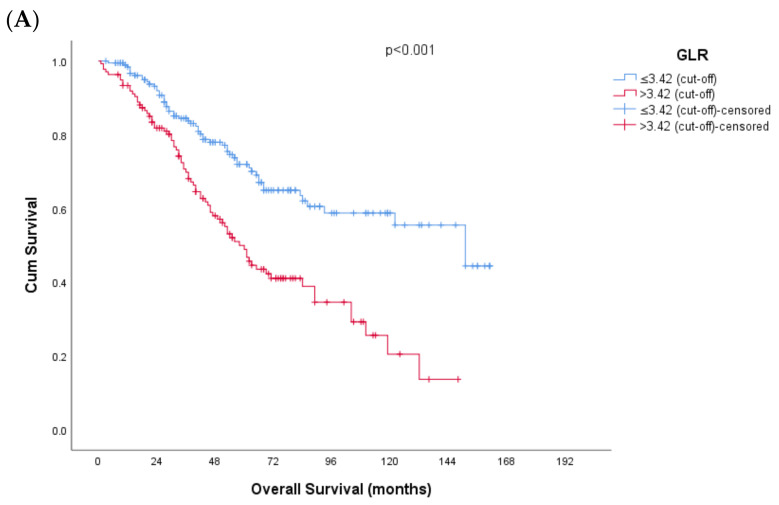

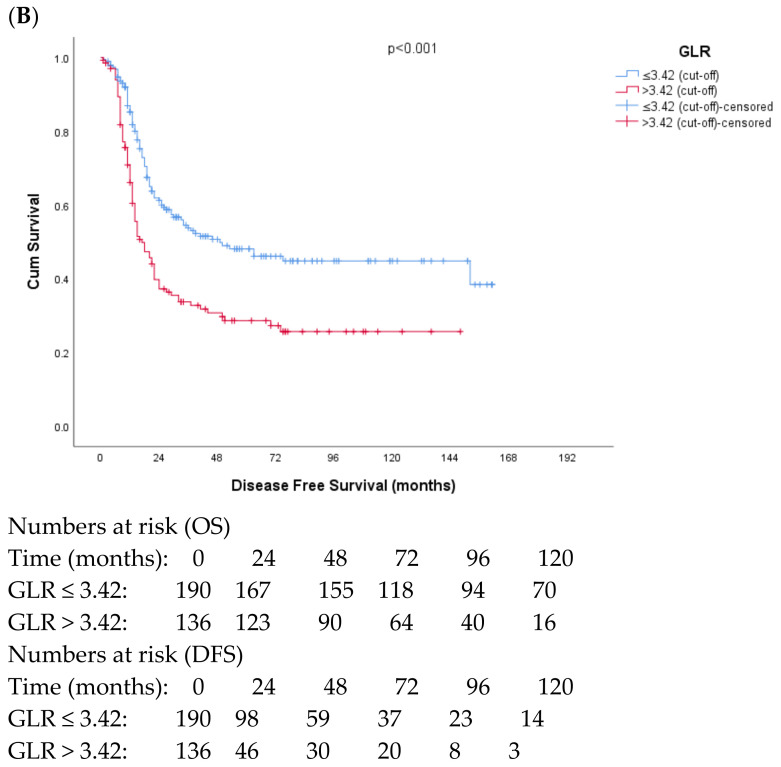
Kaplan–Meier survival curves stratified by GLR cut-off value (≤3.42 vs. >3.42). (**A**) Overall survival. (**B**) Disease-free survival. Numbers at risk are provided below the *x*-axis.

Kaplan–Meier survival curves for overall survival and disease-free survival are presented in Figure 2. The median overall survival (OS) for the entire cohort was 89.0 months (95% CI: 61.8–116.1), and the 5-year OS rate was 61%. Median OS was 151 months (95% CI: 108.4–193.5) in patients with a low GLR score (≤3.42) and 58 months (95% CI: 48.2–67.7) in patients with a high GLR score (>3.42) (*p* < 0.001). Median disease-free survival (DFS) was 27.0 months (95% CI: 18.6–35.3), and the 5-year DFS rate was 39%. The median DFS for patients with a GLR score ≤ 3.42 was 49 months (95% CI: 19.5–78.4), while the median DFS for patients with a GLR score > 3.42 was 17 months (95% CI: 11.7–22.7) (*p* < 0.001).

In univariate Cox regression analysis, high GLR score, age ≥60 years, advanced FIGO stage, tumor grade, and neoadjuvant chemotherapy were identified as significant predictors of reduced OS (Table 2). In multivariate Cox regression analysis, high GLR remained an independent prognostic factor for poor OS (adjusted HR: 1.68, 95% CI: 1.16–2.42, *p* = 0.006).

Analysis of the relationship between GLR and disease progression revealed that disease progression occurred in 29.5% of patients with GLR ≤ 3.42, compared with 55.1% of patients with GLR > 3.42 (χ^2^ = 21.376, *p* < 0.001).

In univariate Cox regression analysis for DFS, high GLR score, advanced FIGO stage, higher tumor grade, and neoadjuvant chemotherapy were identified as significant predictors of reduced DFS. In multivariate Cox regression analysis, a high GLR score remained an independent prognostic factor for shorter DFS (adjusted HR: 1.49, 95% CI: 1.09–2.02, *p* = 0.012). The results of univariate and multivariate Cox regression analyses for DFS are presented in Table 3.

Neoadjuvant chemotherapy status was included in the multivariable Cox regression models to adjust for potential prognostic differences between patients undergoing primary debulking surgery and those receiving neoadjuvant chemotherapy followed by interval debulking surgery. Sensitivity analyses excluding patients with diabetes mellitus were also performed, and the association between GLR and survival outcomes remained consistent. Additional analyses, including models evaluating GLR as a continuous variable and interaction analysis between GLR and neoadjuvant chemotherapy, are provided in the Supplementary Materials (Tables S1–S4).

## 4. Discussion

This study shows that in individuals with epithelial ovarian cancer, an increased GLR at diagnosis is independently associated with both overall survival (OS) and disease-free survival (DFS). Even after adjusting for known clinicopathological factors such as FIGO stage, tumor grade, histological subtype, age, LDH, and albumin levels, the association remained statistically significant.

The findings suggest that GLR may offer prognostic insights that enhance traditional risk categorization metrics. DFS was chosen as the research endpoint to facilitate uniform assessment of outcomes in a cohort of patients who had neoadjuvant chemotherapy followed by interval debulking surgery and those who underwent primary surgery with adjuvant therapy. Since these two treatment strategies involve patient groups with distinct baseline prognostic characteristics, neoadjuvant chemotherapy status was incorporated into the multivariable models to account for potential heterogeneity. Although no clinically meaningful interaction was observed for overall survival, a statistically significant interaction was detected for DFS; however, the magnitude of effect was modest and should be interpreted cautiously given the retrospective design and limited sample size of the neoadjuvant subgroup. This approach aligns with prior studies suggesting that patients receiving primary debulking surgery and those undergoing neoadjuvant chemotherapy may constitute distinct clinical and prognostic subgroups in advanced ovarian cancer [14,15].

In accordance with these findings, an increased GLR has been independently associated with poorer survival outcomes in other malignancies, including cholangiocarcinoma and metastatic renal cell carcinoma [16,17].

GLR may provide complementary prognostic information by integrating both metabolic and immune-related parameters. Conversely, frequently utilized ratios, including the neutrophil-to-lymphocyte ratio (NLR), platelet-to-lymphocyte ratio (PLR), and systemic immune-inflammation index (SII), predominantly indicate systemic inflammation and the redistribution of immune cells [18,19]. Despite their predictive importance in ovarian cancer, meta-analyses have demonstrated significant heterogeneity and a lack of a distinctly superior marker [8,13,20]. In this study, direct comparisons with other inflammatory markers, including NLR, PLR, and SII, could not be performed due to the unavailability of neutrophil and platelet counts.

Consistent with the biological importance of metabolic reprogramming in cancer advancement and treatment resistance, research on other solid tumors has shown that GLR retains independent prognostic value in multivariate models [18,19,20,21,22]. In metastatic gastric cancer, GLR was the only index independently correlated with both overall and progression-free survival, with analogous results observed in pancreatic cancer and extensive pan-cancer cohorts [12,18,21]. Consequently, GLR may serve as an easily accessible adjunct biomarker indicative of metabolic and immune-related host responses, rather than a replacement for established clinicopathological parameters.

Biologically, GLR encapsulates the cumulative impact of tumor-related metabolic dysregulation and host immunological state by synthesizing fasting blood glucose levels and peripheral lymphocyte numbers [21,22]. Hyperglycemia enhances tumor proliferation by increasing aerobic glycolysis and activating proinflammatory signaling pathways, including NF-κB and HIF-1α, which are associated with heightened production of inflammatory cytokines such as IL-6, IL-1β, and TNF-α [7,16,23]. The metabolic and inflammatory alterations lead to aggressive tumor behavior and unfavorable survival outcomes in many solid malignancies [12]. Lymphopenia, on the other hand, signifies compromised antitumor immune surveillance and has been repeatedly linked to worse prognosis in cancer patients [12,22]. By combining these two synergistic elements, GLR operates as a composite biomarker that signifies both hyperglycemia-related tumor aggressiveness and lymphocyte-mediated immunological efficacy, thereby providing a credible rationale for its prognostic significance [21,22,24]. This interpretation aligns with emerging evidence regarding cancer immunometabolism, wherein metabolic changes may influence both tumor progression and host immune function [25,26].

The incorporation of the GLR into prognostic models, in addition to traditional TNM staging, has been demonstrated to improve survival prediction in colorectal cancer [24]. Our findings enhance the prognostic significance of the GLR in epithelial ovarian cancer and endorse its potential as a straightforward and economical biomarker for fundamental risk classification. The current study found that GLR’s discriminatory performance was moderate (AUC = 0.652), which is consistent with the fact that single biomarkers generally have limited discriminatory performance in complex clinical settings. Moderate AUC values suggest that GLR should not be used alone, but it may be more useful when combined with other clinical models [27,28].

Diabetes mellitus is another potential confounder, as it may influence fasting glucose levels independently of tumor biology. Although HbA1c values and detailed antidiabetic medication data were not available in this retrospective cohort, sensitivity analyses excluding diabetic patients yielded consistent results, supporting the robustness of the association between GLR and survival outcomes. Moreover, previous studies have shown that baseline hyperglycemia may adversely affect cancer outcomes, underscoring the importance of considering metabolic comorbidities when interpreting glucose-based prognostic indices [29,30].

This study has several limitations. First, its retrospective single-center design may restrict generalizability, and residual confounding cannot be ruled out. Second, the cohort comprised patients who underwent both primary debulking surgery and neoadjuvant chemotherapy, followed by interval debulking surgery, potentially introducing clinical heterogeneity. Third, the GLR cut-off value was determined through ROC analysis within the same cohort, which raises the potential for overfitting; consequently, validation in independent datasets is necessary to establish the stability and clinical significance of the proposed threshold [31,32]. Although an internal split-sample validation approach was performed, GLR did not consistently retain statistical significance in multivariable models, likely due to reduced statistical power in the smaller validation subset; however, the direction of effect remained consistent. Finally, direct comparisons with other established inflammation-based indices (e.g., NLR, PLR, SII, and PNI) could not be performed due to missing neutrophil and platelet data. Consequently, the findings must be regarded as preliminary and hypothesis-generating; further prospective multicenter studies are necessary to externally validate the prognostic significance of GLR and elucidate its additional utility beyond established clinicopathological variables.

## 5. Conclusions

In this study, the pretreatment GLR was significantly associated with both overall survival and disease-free survival in patients with epithelial ovarian cancer. High GLR values were linked to poorer outcomes and remained an independent prognostic factor in multivariable analyses. Given its low cost and routine availability, the GLR may represent a practical biomarker for basic risk stratification. However, external validation in larger prospective multicenter cohorts is required before the GLR can be implemented in routine clinical practice.

## Figures and Tables

**Table 1:** Baseline clinical characteristics of patients according to GLR groups. Continuous variables are presented as the median (IQR).

Characteristic	Low GLR (≤3.42) (*n* = 190)	High GLR (>3.42) (*n* = 136)	*p* Value
Age, median (IQR)	53.0 (15.8)	58.0 (12.0)	0.004
Glucose (mmol/L), median (IQR)	5.11 (0.778)	6.00 (1.69)	<0.001
Lymphocyte count, median (IQR)	2.02 (0.628)	1.29 (0.500)	<0.001
LDH, median (IQR)	223 (92.5)	253 (132)	<0.001
Albumin, median (IQR)	42.0 (7.75)	37.0 (11.0)	<0.001
Diabetes mellitus, *n* (%)	30 (15.8)	45 (33.1)	<0.001
FIGO stage, *n* (%)			<0.001
I–II	71 (37.4)	21 (15.4)	
III–IV	119 (62.6)	115 (84.6)	
Tumor grade, *n* (%)			0.002
Grade 1	43 (22.6)	13 (9.6)	
Grade 2	18 (9.5)	8 (5.9)	
Grade 3	129 (67.9)	115 (84.6)	
Histology, *n* (%)			0.645
Serous	125 (65.8)	98 (72.1)	
Endometrioid	19 (10.0)	11 (8.1)	
Mucinous	7 (3.7)	1 (0.7)	
Clear cell	18 (9.5)	10 (7.4)	
Adenocarcinoma	12 (6.3)	10 (7.4)	
Papillary serous	8 (4.2)	5 (3.7)	
Other	1 (0.5)	1 (0.7)	
Treatment strategy, *n* (%)			0.012
PDS + adjuvant chemotherapy	175 (92.1)	113 (83.1)	
NACT + IDS	15 (7.9)	23 (16.9)	

Abbreviations: GLR, glucose-to-lymphocyte ratio; FIGO, International Federation of Gynecology and Obstetrics; LDH, lactate dehydrogenase; PDS, primary debulking surgery; NACT, neoadjuvant chemotherapy; IDS, interval debulking surgery.

**Table 2:** Univariate and multivariate Cox regression analysis for overall survival.

Variable	Univariate HR (95% CI)	*p*	Multivariate aHR (95% CI)	*p*
GLR group (>3.42 vs. ≤3.42)	2.30 (1.62–3.27)	<0.001	1.68 (1.16–2.42)	0.006
Diabetes mellitus (Yes vs. No)	1.16 (0.78–1.73)	0.458	0.74 (0.49–1.12)	0.157
Tumor grade				
Grade 3 (reference)	1		1	
Grade 2 vs. Grade 3	0.29 (0.12–0.72)	0.007	0.68 (0.25–1.87)	0.460
Grade 1 vs. Grade 3	0.13 (0.06–0.30)	<0.001	0.43 (0.15–1.22)	0.112
FIGO stage (3–4 vs. 1–2)	6.59 (3.59–12.10)	<0.001	3.13 (1.38–7.11)	0.006
Neoadjuvant chemotherapy (Yes vs. No)	2.57 (1.60–4.15)	<0.001	1.63 (1.00–2.66)	0.048
Age group (≥60 vs. <60)	2.14 (1.52–3.03)	<0.001	1.69 (1.19–2.41)	0.003

aHR, adjusted hazard ratio; CI, confidence interval; FIGO, International Federation of Gynecology and Obstetrics; GLR, glucose-to-lymphocyte ratio; HR, hazard ratio.

**Table 3:** Univariate and multivariate Cox regression analyses for disease-free survival (DFS).

Variable	Univariate HR (95% CI)	*p*	Multivariate aHR (95% CI)	*p*
GLR group (>3.42 vs. ≤3.42)	1.88 (1.40–2.52)	<0.001	1.49 (1.09–2.02)	0.012
Diabetes mellitus (Yes vs. No)	1.01 (0.71–1.44)	0.941	0.69 (0.48–0.99)	0.045
Tumor grade				
Grade 3 (reference)	1		1	
Grade 2 vs. Grade 3	0.27 (0.13–0.58)	0.001	0.41 (0.18–0.93)	0.032
Grade 1 vs. Grade 3	0.12 (0.06–0.24)	<0.001	0.21 (0.09–0.49)	<0.001
FIGO stage (3–4 vs. 1–2)	4.87 (3.08–7.71)	<0.001	1.80 (1.01–3.21)	0.048
Neoadjuvant chemotherapy (Yes vs. No)	3.02 (1.98–4.62)	<0.001	2.15 (1.40–3.31)	<0.001
Age group (≥60 vs. <60)	1.42 (1.05–1.91)	0.023	1.04 (0.77–1.41)	0.794

aHR, adjusted hazard ratio; CI, confidence interval; FIGO, International Federation of Gynecology and Obstetrics; GLR, glucose-to-lymphocyte ratio; HR, hazard ratio.

## Data Availability

The datasets generated and/or analyzed during the current study are available from the corresponding author on reasonable request. The data are not publicly available due to ethical restrictions and patient confidentiality.

## Supplementary Materials

The following supporting information can be downloaded at: https://www.mdpi.com/article/10.3390/jcm15051999/s1, Table S1: Sensitivity analysis excluding patients with diabetes mellitus (OS and DFS); Table S2: Cox regression analysis using continuous GLR (OS and DFS); Table S3: Interaction analysis between GLR and neoadjuvant chemotherapy status (OS and DFS); Table S4: Internal validation of the GLR cut-off using a 70/30 split-sample approach.
