# Supplementary material for: The Pretreatment Glucose-to-Lymphocyte Ratio as an Independent Prognostic Biomarker in Ovarian Cancer
Source: J Clin Med. 2026 Mar 5;15(5):1999. doi: 10.3390/jcm15051999 (PMC12985648; doi:10.3390/jcm15051999)
Supplement: Supplementary file 1 [file jcm-15-01999-s001.zip › jcm-4149355-supplementary.pdf]

Journal of Clinical Medicine

MDPI

SUPPLEMENTARY MATERIALS

The following supplementary analyses were performed to address reviewer concerns regarding potential confounding factors, treatment heterogeneity, and the robustness of the GLR cut-off. Sensitivity analyses excluding patients with diabetes mellitus were conducted to evaluate the stability of the prognostic effect of GLR. Additionally, Cox regression models using GLR as a continuous variable were performed to reduce cut-off dependency. To assess potential effect modification by treatment strategy, an interaction term between GLR and neoadjuvant chemotherapy status was tested. Finally, internal validation of the GLR cut-off was performed using a split-sample approach (70% training and 30% validation cohort).

Supplementary Table S1: Sensitivity analysis excluding patients with diabetes mellitus (OS and DFS).
Supplementary Table S2: Cox regression analysis using continuous GLR (OS and DFS).
Supplementary Table S3: Interaction analysis between GLR and neoadjuvant chemotherapy status (OS and DFS).
Supplementary Table S4: Internal validation of the GLR cut-off using a 70/30 split-sample approach.

Supplementary Table S1. Sensitivity analysis excluding diabetic patients

Overall survival (OS)

Variable	Univariate HR (95% CI)	p value	Multivariate aHR (95% CI)	p value
**GLR group (>3.42 vs ≤3.42)**	2.31 (1.55–3.45)	<0.001	1.71 (1.13–2.58)	0.011
**Age (≥60 vs <60)**	2.05 (1.37–3.06)	<0.001	1.50 (0.99–2.28)	0.054
**FIGO stage (III–IV vs I–II)**	8.38 (4.02–17.47)	<0.001	4.34 (1.52–12.41)	0.006
**Neoadjuvant chemotherapy (Yes vs No)**	2.79 (1.56–4.99)	0.001	1.90 (1.04–3.46)	0.036
**LDH (continuous)**	1.00 (1.00–1.00)	0.004	1.00 (1.00–1.00)	0.589
**Albumin (continuous)**	0.95 (0.92–0.97)	<0.001	0.95 (0.93–0.98)	0.001

Disease-free survival (DFS)

Variable	Univariate HR (95% CI)	p value	Multivariate aHR (95% CI)	p value
**GLR group (>3.42 vs ≤3.42)**	1.92 (1.37–2.68)	<0.001	1.51 (1.07–2.14)	0.020
**Age (≥60 vs <60)**	1.39 (0.98–1.97)	0.063	0.90 (0.63–1.29)	0.569
**FIGO stage (III–IV vs I–II)**	6.15 (3.64–10.41)	<0.001	2.58 (1.26–5.26)	0.009
**Neoadjuvant chemotherapy (Yes vs No)**	2.85 (1.70–4.78)	<0.001	2.13 (1.23–3.69)	0.007
**LDH (continuous)**	1.00 (1.00–1.00)	<0.001	1.00 (1.00–1.00)	0.380
**Albumin (continuous)**	0.95 (0.93–0.98)	<0.001	0.97 (0.95–1.00)	0.036

Abbreviations: aHR, adjusted hazard ratio; CI, confidence interval; DFS, disease-free survival; FIGO, International Federation of Gynecology and Obstetrics; GLR, glucose-to-lymphocyte ratio; HR, hazard ratio; LDH, lactate dehydrogenase; OS, overall survival.

https://doi.org/10.3390/xxxxx

Supplementary Table S2. Continuous GLR Cox regression analysis (DM excluded)

Overall survival (OS)

Variable	Univariate HR (95% CI)	p value	Multivariate aHR (95% CI)	p value
**GLR (continuous)**	1.23 (1.13–1.33)	<0.001	1.11 (1.01–1.21)	0.028

Disease-free survival (DFS)

Variable	Univariate HR (95% CI)	p value	Multivariate aHR (95% CI)	p value
**GLR (continuous)**	1.26 (1.16–1.37)	<0.001	1.14 (1.04–1.24)	0.004

Abbreviations: aHR, adjusted hazard ratio; CI, confidence interval; DFS, disease-free survival; GLR, glucose-to-lymphocyte ratio; HR, hazard ratio; OS, overall survival.

Supplementary Table S3. Interaction analysis between GLR and neoadjuvant chemotherapy

Overall survival (OS)

Variable	B	SE	Wald	p value	HR (Exp(B))	95% CI
**GLR × Neoadjuvant interaction term**	0.049	0.095	0.265	0.607	1.050	0.871–1.265

Disease-free survival (DFS)

Variable	B	SE	Wald	p value	HR (Exp(B))	95% CI
**GLR × Neoadjuvant interaction term**	0.176	0.080	4.791	0.029	1.192	1.019–1.396

Abbreviations: CI, confidence interval; DFS, disease-free survival; GLR, glucose-to-lymphocyte ratio; HR, hazard ratio; OS, overall survival.

Supplementary Table S4. Internal validation using a 70/30 split-sample approach

Training cohort (70%)

Outcome	GLR group (>3.42 vs ≤3.42) Univariate HR (95% CI)	p value	Multivariate aHR (95% CI)	p value
**OS**	2.25 (1.48–3.42)	<0.001	1.28 (0.75–2.18)	0.360
**DFS**	2.08 (1.45–2.98)	<0.001	1.63 (0.98–2.71)	0.061

Validation cohort (30%)

Outcome	GLR group (>3.42 vs ≤3.42)	Univariate HR (95% CI)	p value	Multivariate aHR (95% CI)	p value
**OS**	2.32 (1.20–4.47)		0.012	2.06 (0.75–5.65)	0.162
**DFS**	1.49 (0.88–2.50)		0.137	1.27 (0.58–2.77)	0.553

Abbreviations: aHR, adjusted hazard ratio; CI, confidence interval; DFS, disease-free survival; GLR, glucose-to-lymphocyte ratio; HR, hazard ratio; OS, overall survival.
